# Lysosomal Pathogenesis of Parkinson’s Disease: Insights From LRRK2 and GBA1 Rodent Models

**DOI:** 10.1007/s13311-022-01290-z

**Published:** 2022-09-09

**Authors:** Mattia Volta

**Affiliations:** Institute for Biomedicine, Eurac Research - Affiliated Institute of the University of Lübeck, via Volta 21, Bolzano, 39100 Italy

**Keywords:** LRRK2, Glucocerebrosidase, Lysosomes, Parkinson’s disease, Animal models, Neuropathology

## Abstract

The discovery of mutations in LRRK2 and GBA1 that are linked to Parkinson’s disease provided further evidence that autophagy and lysosome pathways are likely implicated in the pathogenic process. Their protein products are important regulators of lysosome function. LRRK2 has kinase-dependent effects on lysosome activity, autophagic efficacy and lysosomal Ca^2+^ signaling. Glucocerebrosidase (encoded by *GBA1*) is a hydrolytic enzyme contained in the lysosomes and contributes to the degradation of alpha-synuclein. PD-related mutations in LRRK2 and GBA1 slow the degradation of alpha-synuclein, thus directly implicating the dysfunction of the process in the neuropathology of Parkinson’s disease. The development of genetic rodent models of LRRK2 and GBA1 provided hopes of obtaining reliable preclinical models in which to study pathogenic processes and perform drug validation studies. Here, I will review the extensive characterization of these models, their impact on understanding lysosome alterations in the course of Parkinson’s disease and what novel insights have been obtained. In addition, I will discuss how these models fare with respect to the features of a “gold standard” animal models and what could be attempted in future studies to exploit LRRK2 and GBA1 rodent models in the fight against Parkinson’s disease.

**Supplementary Information:**

The online version contains supplementary material available at 10.1007/s13311-022-01290-z.

## Introduction

The etiology of Parkinson’s disease (PD) has been a major mystery throughout the 200 years of clinical and scientific investigations on this devastating neurological disorder. For decades, the definition of PD has been “idiopathic”, a term that indicates a disease occurring spontaneously or from an unknown cause. To this day, idiopathic PD (iPD) represents the most common form of the disease. It is characterized by the progressive loss of dopamine (DA) neurons in the substantia nigra pars compacts (SNc) and the widespread accumulation of proteinaceous intraneuronal inclusions termed Lewy bodies (LBs) and neurites (LNs), mainly composed of alpha-synuclein (aSyn) [1, 2]. Continuous efforts have been made to understand the etiology of the disease with several hypotheses having been developed. In the 1980s, the environmental hypothesis took the spotlight, as toxicants were demonstrated to cause nigral neurodegeneration and parkinsonism, propelled by the discovery of the neurotoxin MPTP [3, 4]. In accordance, the onset of parkinsonism was associated with exposure to several environmental factors, such as pesticides, that have been confirmed as neurotoxic in the laboratory [5]. However, a clear causal relationship has been difficult to unequivocally demonstrate due to the challenges posed by population studies, and the number of variables they intrinsically bear.

Interest in the environmental hypothesis was reduced when an unpredicted turn of events at the end of the 1990s revealed the first gene mutation. Mutation in the *SNCA* gene encoding aSyn itself was causally linked to a familial form of PD [6], marking the beginning of the era of genetics in PD [7]. In the following years, several genes were identified which cause both autosomal dominant and recessive forms of familial PD, giving unprecedented molecular insight into disease etiology [8]. Indeed, the discovery of gene mutations and alterations in *Parkin*, *PINK1* and *DJ-1* (with recessive inheritance) highlighted a role for mitochondria biology in nigral neurodegeneration [9], that is also targeted by environmental toxicants. On the other hand, autosomal dominant familial PD linked to *SNCA*, *LRRK2*, *VPS35* provided a window for intracellular vesicle dynamics as a process with pathogenic relevance. While a detailed discussion on vesicle dynamics is beyond the scope of this review (and the reader is directed to excellent literature on the topic, such as [10, 11]), I will here focus on lysosomal biology and its implications in PD onset, progression and potential therapy.

## Lysosomal Function and Parkinson’s Disease

The involvement of lysosomes in PD emerged from two main observations:

- neuropathological evidence indicated alterations in lysosomal markers in patient brains [12, 13];
- the protein products of several genes linked to PD are implicated in lysosomal biology [14].

The latter observation is illustrated by a multitude of reports showing evidence of how LRRK2, aSyn, VPS35, Glucocerebrosidase (GBA1), ATP13A2 impact lysosome function and the alterations produced by pathogenic mutations.

Lysosomes are double-membrane vesicles with an acidic internal pH that allows activity of the several types of digestive enzymes contained in them. Their most studied cellular function is the degradation of cellular and extracellular material that is delivered to it. The catabolic function as downstream effector of the autophagy pathways has been (and still is) a major focus of research in both basic biology and disease-oriented studies. Lysosome-mediated autophagy has been studied in the context of nutrient deprivation, where this catabolic pathway is strongly activated to provide the cell with basic life molecules (e.g., aminoacids) that are thus recycled [15]. In this context, the autophagy-lysosome pathway (ALP) has been mainly viewed as a cell-coping mechanism that comes into play in conditions of threatened survival. Nevertheless, continuous investigations on the basic biology mechanisms highlighted the existence of several subtypes of ALP that can be directed at specific cargoes to be degraded. A notable example is aggrephagy that targets proteins with propensity to form insoluble aggregates (for a comprehensive review on selective ALP see [16]). This function provides an interesting link with neurodegenerative diseases of the aging that are characterized by progressive accumulation of intracellular and extracellular inclusions of proteins that define each disease [17, 18].

In the case of PD, the discovery of disease-causing mutations and variations in the *SNCA* gene, and the contemporary observation that the encoded protein, aSyn, is the main component of pathological inclusions, were a prelude to the seminal discovery that mutant aSyn impairs autophagic degradation [19]. Since then, intense efforts have been dedicated to the understanding of the role of ALP in aSyn neuropathology, and the role played by familial PD-linked proteins in lysosome biology.

As mentioned, aSyn is both a substrate and a regulator of lysosomal function. More recent evidence indicates that aSyn degradation (operated by chaperone-mediated autophagy; CMA) is regulated by LRRK2, mutations of which impair this process [20]. The following decade produced a wealth of knowledge on the interaction of these critical PD proteins in the ALP. Indeed, LRRK2 plays a kinase-dependent role in the onset and spread of aSyn, as evidenced in vitro and in vivo [21–23]. The mechanism whereby aSyn modulates the ALP is still unclear, but is thought to involve a “clogging” of the degradative processes [24]. A vicious cycle is hypothesised to occur, where aggregation-prone aSyn impairs lysosomal degradative capacity, which in turn further reduces the ability of the cell to clear the aggregates.

### Genetics Inspire Biology: LRRK2 and GBA1

At variance with aSyn research, scientific work around LRRK2 has been more specifically directed at identifying the molecular players involved in ALP regulation, and their impact on aSyn accumulation. Here I will summarize these studies for the purpose of this article, while the reader can refer to several reviews in the literature for deeper insights (e.g., [25–29]).

**Fig. 1:**
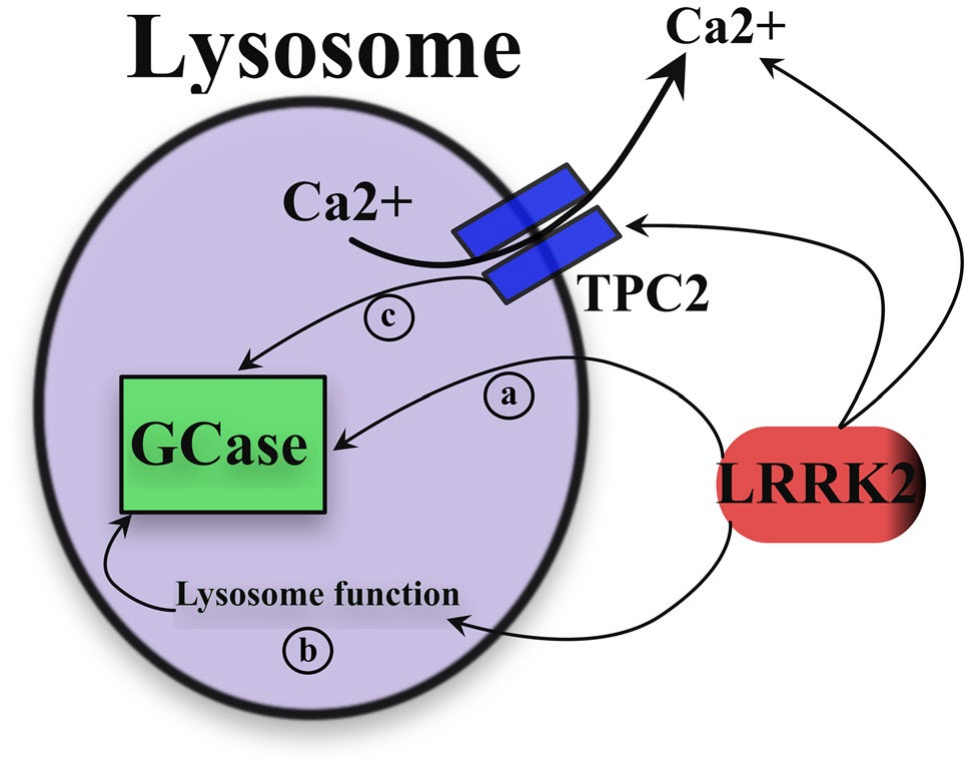
Interactions of LRRK2 and GCase in lysosome function. LRRK2 modulates lysosome function, probably through regulation of Ca^2+^ release from this store. Lysosomal Ca^2+^ exits in the cytosol through TPC2 channels, whose efficacy is altered by LRRK2 mutations. Glucocerebrosidase (GCase) is trafficked into the lysosomal lumen where it is activated by the acidic pH. LRRK2 is capable of modulating GCase activity, but the exact molecular mechanism is yet to be elucidated, as no indications exist whether LRRK2 might be present inside the lysosome. LRRK2 might directly affect GCase activity (**a**); or it could alter lysosome function, with downstream consequences on GCase biology, such as activity, trafficking or expression (**b**); LRRK2-dependent regulation of lysosomal TPC2 channels could also lead to alterations in GCase functionality as a secondary consequence (**c**)

In the early days of LRRK2 biology research, it was already noted that LRRK2 might functionally interact with aSyn (and other pathology-related proteins) [30]. First, PD patients carrying LRRK2 mutations present a varying degree of neuropathologies, despite a similar clinical presentation [31, 32], suggesting a role for LRRK2 in proteinopathy. Also, LRRK2 and aSyn were proposed to function in intersecting pathways in molecular and pathological contexts [33–35]. These observations prompted the investigation of LRRK2 functionality in the ALP, as it was found that LRRK2 physiologically has a propensity to localize to membranous structures in cells [36–38]. Consistently, LRRK2-dependent effects have been reported on macroautophagy, where the formation of double-membrane cargo engulfing autophagosomes might be modulated by LRRK2 kinase activity [27]. However, a clear direction of modulation and the precise alterations bore by PD-linked mutations in LRRK2 proved extremely difficult to unequivocally demonstrate [39, 40]. To this date, whether LRRK2 kinase activity is directly or inversely proportional to autophagosome formation and the autophagic flux remains a matter of debate. On the other hand, studies are almost completely concordant that hyperactive LRRK2 kinase impairs lysosome function, pointing to a more evident impact on lysosome biology. LRRK2 is recruited to stressed lysosomes, bringing its substrates along on the lysosome membrane [41]. The lysosomal localization supported the role of LRRK2 in CMA [20], an ALP subtype independent of autophagosomes. In addition, LRRK2 has been reported to modulate lysosomal two-pore channel 2 (TPC2). These channels are involved in Ca^2+^ release from lysosomes, a process recently clarified as required for correct lysosomal proteolysis and induction of autophagy [42]. Specifically, Hockey and colleagues found that fibroblasts from LRRK2 PD patients carrying the G2019S mutation (that enhances kinase activity) had exaggerated TPC2 Ca^2+^ release, impacting lysosome morphology and function [43] (Fig. 1). It is thus possible to hypothesize that LRRK2 primary work at lysosomes is in fine regulatory processes, such as Ca^2+^ dynamics, that impact downstream proteolysis and ALP.

Our group also observed that LRRK2 kinase activity mildly affects autophagic flux in cells, while more potently modulating lysosomal biology with a direct impact on aSyn handling [21]. Consistently, previous studies in vivo indicated that G2019S-LRRK2 augments aSyn accumulation in mouse models of neuropathology based on viral delivery of aSyn or inoculation of synthetic fibrils [22, 44]. As mentioned earlier, pharmacological inhibition and gene silencing are effective approaches to counteract aSyn neuropathology in the preclinical setting and are currently under clinical trials in humans (e.g., ClinicalTrials.gov identifiers: NCT03976349; NCT05348785).

A critical advance in the appreciation of the role of lysosomes in PD pathogenesis came when mutations in the *GBA1* gene were recognized to dramatically increase the risk of developing PD [45]. *GBA1* encodes for the lysosomal hydrolase glucocerebrosidase (GCase), which metabolizes glucosylceramide into glucose and ceramide. Homozygous or compound heterozygous mutations in *GBA1* cause Gaucher’s disease (GD), an autosomal recessive lysosomal storage disorder that can present with neurological symptoms [46]. The pathological hallmark of GD is the presence of Gaucher cells, macrophages in which aberrant lysosomes are heavily present and contain large amounts of undigested glycolipids (such as glucosylceramide and glucosylsphingosine), directly relating the lysosomal abnormalities to *GBA1* mutations and loss of function [46, 47]. GD patients have a higher risk of developing PD [48], and carriers of heterozygous mutations bear an ~ five-fold increase in PD risk [45, 49]. However, *GBA1* mutations are a risk factor for PD and not disease-causing mutations, as not every carrier will develop the disorder [45, 46]. The reasons for this reduced penetrance are still unclear and it will be interesting to understand its biological bases, given the importance of the cellular process affected.

*GBA1* mutations in PD patients brought the spotlight once again on the centrality of lysosomes in the pathogenesis and pathology of PD. Most importantly, GCase was shown to process aSyn in the lysosomes and constitute a negative feedback loop. Reduction of GCase activity enhances the accumulation of aSyn, that in turn further inhibits GCase [50], producing a “chicken-and-egg” situation that might suggest that the initial insult could vary, but lead to the same disease. Indeed, different *GBA1* mutations linked to PD impair GCase activity through different mechanisms, that are exploited for therapeutic approach design [51]. Consistent with this view, small molecule chaperones that produce activation of GCase appear to reduce aSyn pathological burden in culture and rodents [52, 53]. The clinical use of GCase activators might be a promising therapy as reduced GCase was observed in iPD patient brains, in the absence of *GBA1* mutations [54, 55]. This was not replicated in peripheral samples in a recent study [56], possibly indicating that GCase activity in the brain could be under a specific regulation, hampering its diagnostic value but not the therapeutic value.

### The Functional Interaction of LRRK2 and GBA1

The discovery, and subsequent biological characterization, of several genes linked to familial PD hinted that a few cellular processes might be of particular relevance for pathogenesis, as the protein products of those genes appear to cluster around said processes. Indeed, vesicle trafficking, endosome regulation and ALP are the focus of a great number of these PD genes [8]. Thus, the study of functional interactions between them flourished and contributed to extremely interesting observations. This is the case for *LRRK2* and *GBA1* as well, supported by their common effects on lysosome biology (Fig. 1). In DA neurons derived from PD patient cells, the activity of GCase and LRRK2 kinase was inversely proportional, with LRRK2 inhibition leading to increased GCase function in neurons from either *LRRK2* or *GBA1* mutation carriers, but also from control cells [57]. This indicates that LRRK2-mediated regulation of GCase could be a physiological mechanism, being disrupted by PD-linked mutations. The study did not further explore the pathogenic consequences of such interaction, aside from downstream reduction of pS129-aSyn and oxidized DA, considered readouts of neuronal toxicity. These results found recent in vivo confirmation, as GCase hydrolytic capacity was enhanced in the striatum of LRRK2 knock-out (KO) and kinase-dead (KD) mice. However, no changes in striatal GCase activity were found in G2019S-LRRK2 knock-in (KI) animals [58]. Given potential variability across models (human neurons vs rodent brain tissue), the possibility exists that LRRK2-GCase interaction might be differentially regulated in different neuronal populations or brain areas [59], somewhat consistent to regional vulnerability of neurodegeneration and neuropathology [60]. However, findings in *GBA1* mutant astrocytes indicate that LRRK2-mediated GCase regulation impacts non-neuronal systems and potentially modulates immune functions [61]. This apparently straightforward picture is complicated by clinical observations in dual *LRRK2*-*GBA1* mutation PD patients, who seem to have a milder motor and non-motor symptomatology compared to *GBA1* PD and iPD, but closer to LRRK2 PD (recently reviewed in [62]). Here the authors conclude that LRRK2 mutations might have a modifying role in *GBA1* PD patients, attenuating the clinical picture. Nevertheless, the critical caveat of the very low number of dual mutation carriers that have been examined so far must be kept in mind when drawing conclusions.

## LRRK2 Rodent Models and Lysosome Biology

**Fig. 2:**
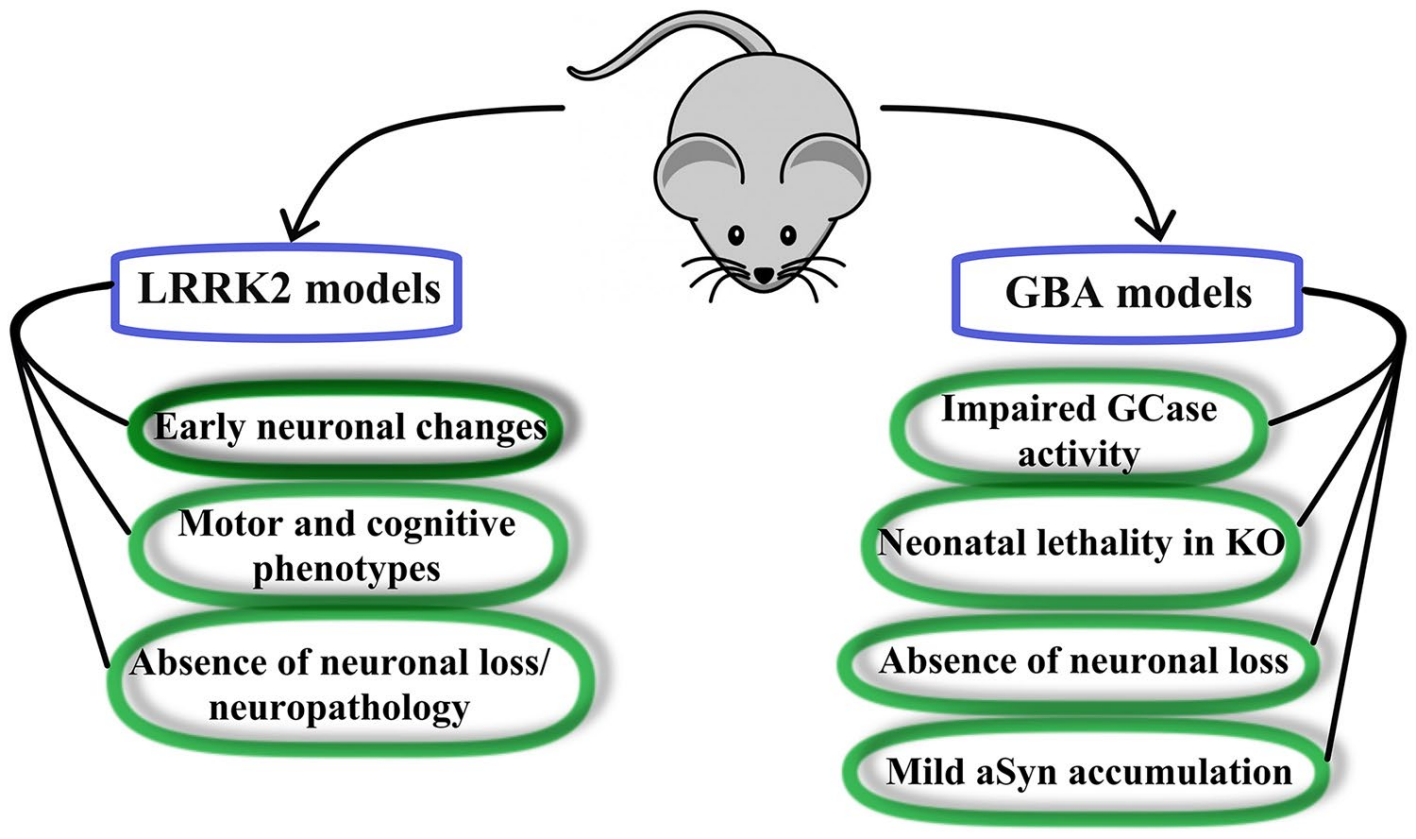
Main features of LRRK2 and GBA1 knock-in mice. Mice carrying PD-related mutations in the endogenous *Lrrk2* gene do not display neurodegeneration or neuropathology, but show alterations in neuronal and synaptic function reminiscent of early-stage PD. This is generally accompanied by changes in motor and cognitive abilities, with reports of age-dependency of these phenotypes. Similarly, KI mice with *GBA1* mutations do not show neuronal loss and generally only mild accumulation of aSyn. Molecularly, they correctly present reduction of GCase enzymatic activity in several tissues, but behavioral phenotypes have been scarcely investigated. Of note, constitutive GCase loss of function is neonatally lethal in mice

Following the discovery of PD pathogenic mutations in LRRK2 [32, 63], a vast number of genetically modified animal models have been developed, with the mouse taking the center stage among different species. These models have been engineered in various ways, deleting the murine *Lrrk2* gene (KO), expressing the human or murine *Lrrk2* via cDNA and BAC, or introducing PD mutations in the murine genome (KI). Phenotyping has mostly focused, especially in the early years, on histology, motor phenotypes and neurophysiology (Fig. 2) with the aim of providing predictive validity for preclinical drug testing. The reader can refer to several authoritative reviews regarding these studies in LRRK2 animals (e.g., [39, 40, 64–66]). In this article, I will focus the attention on insights toward lysosomal biology and dysfunction gathered from LRRK2 rodent models, a section of the LRRK2 field that still needs deep exploration.

### LRRK2 KO Rodents

Animals carrying deletions of the *Lrrk2* gene have been quickly developed to understand the physiological role of LRRK2. Initial studies did not investigate alterations to the ALP/lysosome function in the brain [67–69]. However, other reports indicated autophagic alterations, abnormal lysosomal accumulation and altered lysosomal trafficking in peripheral organs, such as lungs and kidneys, of KO mice [70–72]. Similarly, pharmacological inhibition of LRRK2 kinase activity causes tissue alterations in lungs and kidneys. Specifically, type II pneumocytes of the lungs display accumulation of lamellar bodies, which are secretory lysosome-related organelles, in mice and monkeys treated with LRRK2 inhibitors. However, distinctly from KO animals, lysosome-related renal pathology is not readily identified in preclinical testing of LRRK2 inhibitors (for a comprehensive review, see [73]). Strikingly, renal alterations in autophagy appeared to follow a biphasic course when mice were analyzed at different ages [74]. In accordance with these results, following studies in mice and rats carrying *Lrrk2* deletions also reported the absence of lysosomal alterations in the brain, while renal autophagy was variously affected [75, 76], providing confirmation of findings in peripheral organs, which are now an accepted phenotype of KO animals. It is also important to note here that most of these observations displayed considerable size effects, especially in the analyses of protein levels (as examples, see [67, 71, 74]). Coupling effect size to replication by different groups provides a certain degree of confidence in the phenotype to be accepted as true. The discrepancy between the brain and the periphery has been attributed to the different expression levels of LRRK2 in these organs. Indeed, LRRK2 is physiologically expressed at higher levels in the kidney with respect to the brain [77], thus probably rendering these organs more susceptible to LRRK2 loss. On the other hand, it was also hypothesized that the homolog LRRK1, whose expression is relatively abundant in brain tissue [78], could compensate for LRRK2 loss of function in the brain. Indeed, double LRRK1/LRRK2 KO mice showed age-dependent loss of nigral DA neurons and accumulation of autophagic vacuoles and p62 in the same area [79]. Replication and expansion of these results in double KO animals from independent laboratories is awaited to shed further light on the neuronal function of the LRRK homologs and their functional interdependence. Even though LRRK2 loss of function has not been reported to be associated with PD [80], the relevance of these results must not be undermined as LRRK2 inhibitors are being tested in the clinic and it is important to learn about potential side effects, both in the periphery and at central level.

### Transgenic LRRK2 Rodent Models

In order to model LRRK2 PD, mice and rats have been engineered to express human or murine LRRK2, in either WT or mutant forms. As mentioned above, studies have mostly focused on reproducing PD pathophysiology and neuropathology, and functional investigations of ALP in vivo are not yet abundant. Nevertheless, the literature does contain important indications on this matter.

Transgenesis using cDNA-based expression constructs has been extensively employed to ensure robust overexpression. Most of these models do display neurodegeneration, albeit not recapitulating the genetic etiology of LRRK2 PD [81]. A handful of reports investigated ALP phenotypes in these mice. An early study showed accumulation of autophagic vacuoles in cortex and striatum of G2019S-LRRK2 and, to a lesser extent, R1441C-LRRK2 overexpressing mice, albeit in the absence of aSyn or Tau neuropathologies [82]. More recently, ALP markers such as LC3 and p62, along with aSyn, were reported to be quite largely increased in G2019S-LRRK2 transgenic (TG) animals [83]. Thus, indications from these models are currently inconclusive, with regard to lysosome biology, and few replication studies are present in the literature. From these, we can infer possible impairment of ALP, given the accumulation of vacuoles and markers, in mutant TG animals.

The development of TG animals based on the Bacterial Artificial Chromosome (BAC) surpassed a few of the drawbacks of cDNA transgenesis, such as mirroring the physiological expression pattern. On the other hand, these genetic models mostly do not display neurodegeneration nor aSyn neuropathology. Nevertheless, they have been put to good use to identify biological and neuronal processes affected by LRRK2 [66]. However, only a recent article investigated ALP in BAC LRRK2 rats, where authors have noted > two-fold increase of LC3-II levels in brain tissue from R1441C-LRRK2, but not G2019S-LRRK2 BAC rats, somewhat consistent with impaired ALP suggested by cDNA TG animals. In the same report, the authors provide a great deal of results showing lysosomal alterations in primary cultures derived from the same animals, but direct observations in vivo or in tissue are not available [84]. Of note, BAC WT-LRRK2 mice were utilized in a model of colitis to obtain bone marrow-derived cells, which display autophagy suppression as p62 accumulates [85]. In this case as well, no direct observations in the animals have been performed. Altogether, information on ALP functionality in BAC models is not fully established and most results have not been replicated, leaving an open question on lysosome phenotypes in these animals.

### LRRK2 KI Animals

The introduction of PD-linked mutations in the murine *Lrrk2* gene led to the development of etiologically relevant animal models that do not carry confounds of transgene overexpression [64]. Despite the general lack of neurodegeneration and neuropathology (aside some mild Tau and oligomeric aSyn accumulations [64, 66]) KI mice are considered useful to study prodromal disease and the cellular processes that might be involved in pathogenesis. An early report found alterations in the ALP-related mTOR signaling in the kidneys of G2019S-LRRK2 KI mice, but no experiments in brain tissue were shown [71]. Baseline mTOR signaling changes were not observed in striatal tissue of the same line of mice in a more recent study. Instead, increases in p62 and mTOR levels, and a reduction of p-mTOR were induced in KI animals, but not WT, upon pharmacological LRRK2 inhibition [58]. Of note, increased pS129-aSyn levels were reported in the striatum of these animals [58, 86]. At variance, a different line of G2019S-LRRK2 KI mice displayed mildly increased striatal levels of LC3-II, and no changes in the LC3-II/LC3-I ratio [87]. Elsewhere, LC3-I was markedly reduced in the cortex of the same G2019S-LRRK2 KI mouse line, along with reduced levels of the lysosomal protein LAMP1 (lysosome-associated membrane protein 1) [88]. Here it is important to highlight that harmonized guidelines on autophagy investigations urge to evaluate LC3 conversion (and also other ALP markers, such as p62 levels) also upon pharmacological inhibition of lysosomal activity to more accurately evaluate autophagic flux [89]. While this is a routinary approach in cellular cultures, such manipulations in vivo bear significantly increased challenges (as most compounds are toxic and unspecific), and consequently studies including this paradigm are few and far between. Comparisons between different studies are complicated by the different KI lines that have been developed, which seem to yield as yet unclarified differences. The replication of specific results (e.g., variations in LC3-II levels or conversion) appear challenging and unanimous conclusions cannot be drawn at the moment.

At variance with G2019S-LRRK2 mutants, a recent study in R1441G-LRRK2 KI mice reported more profound alterations in CMA, with age-dependent accumulations of striatal LAMP2A and GAPDH (a CMA substrate), and lysosomal clustering in the same area. These changes were accompanied by accumulation of oligomeric aSyn, but not overt neuropathology nor neuronal loss [90], further stressing the subtlety of the phenotypes exhibited by KI animals. These specific observations have not yet been replicated in other R1441G KI animals.

The complicated view of ALP regulation by LRRK2, especially in vivo, has been recently challenged by the observations that G2019S-LRRK2 KI mice display altered mitophagy, but unchanged autophagy, in both the brain and peripheral organs with high LRRK2 expression. Interestingly, in brain tissue mitophagic changes appeared area-specific, affecting SNc and cortex but not cerebellum [91]. Altogether, these data could imply that LRRK2 and its mutations, more consistently affect lysosome biology. This would impact CMA and mitophagy, which do not rely on autophagosome formation, providing a novel view that might add consistency between studies. However, in opposition to this conclusion is the elegant demonstration that increased LRRK2 kinase function disrupts the transport of autophagosomes along neuronal axons [91]. A caveat to consider is that the latter evidence was mostly gathered from primary neuronal cultures and not in vivo. Nevertheless these two views could still coexist. Indeed, a differential regulation of somatic vs synaptic autophagy is gaining consideration, with LRRK2 playing crucial roles in the regulation of ALP at the synaptic terminal [14, 92]. We could speculate that the compartmentalization of ALP might be linked to different regional localization of its regulators, including LRRK2.

## Rodent Models of GBA1-PD and Lysosome Biology

Efforts to generate mouse models of GCase dysfunction date back to the 1970s, as researchers worked to develop therapies for GD [93]. As is the case for several diseases, animals were initially based on chemical treatments. This evolved to genetic *GBA1* models beginning to appear in the 1990s [94]. In this section, I will analyze rodent models carrying *GBA1* genetic manipulations with the scope of discussing its role in lysosome function and with a preferred view for models applicable to PD research (Fig. 2). It is worth noting that these modeling efforts (and hence their discussion depth) is challenged by the nearly 300 mutations found in *GBA1* [46, 47].

### GBA1 Downregulation and Point Mutations

The first *GBA1* KO mouse was reported in 1992 with the aim of genetically modeling GD. Despite the successful, dramatic reduction in GCase activity and the pathological observations in different organs, the KO had neonatal lethality, strongly limiting its preclinical usefulness [94]. This led researchers to focus efforts on introducing GD-linked point mutations. Notwithstanding a large reduction in GCase activity in various tissues generally, most mutant mice did not display gross phenotypic abnormalities [47]. Also, many of these mutations did not lead to the same level of GCase inhibition in the brain (which remained higher compared to other organs) making it challenging to model neurological signs of GD. A notable exception is the N370S mutant mouse, which died within 24h from birth and shared many features of the *GBA1* KO mouse [95]. To overcome these difficulties, in 2006 conditional mouse models began to appear. The post-natal genetic manipulation allowed by Cre-mediated recombination improved the survival of the animals and modeled peripheral features of GD. The involvement of the CNS and neurological symptoms of GD however remained limited [47]. This led to further efforts of conditionally modulating *GBA1* expression in the brain. These approaches did lead to reduced GCase function in the CNS and development of neurological abnormalities. This occurred very early in the life of the mice (7–16 days after birth) culminating in paralysis [96] and thus still presented important limitations to preclinical investigations. Similarly, GD mutations were introduced under the control of the Cre recombinase to induce their expression in the CNS, leading to phenotypes overlapping to the conditionally deleted *GBA1* mice [97].

### GBA1 Manipulations to Model Parkinson’s Disease

The discovery of *GBA1* mutations as prominent risk factors for PD prompted the investigation of *GBA1* mouse models for PD pathophysiology and drug validation (Fig. 2). Thus, initial studies used available GD models to assess behavioral and histological phenotypes related to PD. However, this approach is riddled with caveats. First, the reduction of GCase activity is not predictive of PD. Second, the frequency of PD in heterozygote and homozygote *GBA1* mutation carriers is similar. Nevertheless, many more PD patients carry mutations in heterozygosis. As discussed above, the genetic *GBA1* models of GD rely on gene deletion or homozygote mutations, significantly differing in the representation of etiology (see the excellent review by Farfel-Becker and colleagues for a complete roundup of this issue [98]).

The studies that followed and tried to address GBA1-PD more specifically focused on milder degrees of GCase impairment, mostly through mutation heterozygosis. In this view, the L444P mutation is commonly found in PD patients [45] and heterozygote KI mice have been developed. These animals do display cellular alterations relevant for PD such as, autophagy and mitophagy abnormalities together with increased aSyn levels. However, they do not develop neuropathology or neurodegeneration even at older ages [99, 100]. To avoid the lethality in L444P/L444P KI, this mutation has also been inserted, in homozygosis, under the control of the Cre-Lox recombinant system. This conditional mouse had longer lifespan and increased aSyn in the striatum, although this increase was not quantified. In addition, no deep phenotyping has been carried out, limiting the appreciation of its usefulness in PD modeling [101].

The N370S mutation is commonly found and associated to a milder clinical presentation [98], but surprisingly is neonatally lethal in mice when present in homozygosity [47]. Models of N370S heterozygote mutation have been mainly studied in combination with aSyn insults, and will be discussed in the following section.

The D409H substitution is a rare mutation in PD patients, but a severe one [102]. However, KI mice (both hetero- and homozygous) do not exhibit PD histopathology nor behavioral phenotypes [103].

In the same residue, the D409V mutation is of peculiar interest. This substitution is not found in PD patients (and is rarely present, in heterozygosis, in GD patients) [104, 105], but it has functional implications on GCase biology. KI mice have strong decreases in brain GCase activity, accompanied by aSyn accumulation in the hippocampus and memory deficits [106, 107]. Unfortunately, these results were not replicated in independent studies [108–110]. These discrepancies, together with the lack of etiological relevance of D409V, hampers the translational utility of these models.

### Animal Models Combining GBA1 and SNCA Manipulations

The lack of prominent aSyn pathology in *GBA1* mutant mice prompted the attempt to combine *GBA1* mutation KIs with aSyn TG animals or aSyn viral delivery, to appreciate whether GCase impairment could synergize with aSyn. Indeed, L444P heterozygotes do not display PD phenotypes (see above), but enhanced motor deficits and induced earlier hippocampal pS129-aSyn accumulation in A53T-aSyn mice [111]. Similarly, KI of L444P synergized with AAV-aSyn treatment-induced nigral DA neuron loss, with no appreciable differences in pS129-aSyn neuropathology in SNc [112]. Consistently, A53T-aSyn TG mice crossbred with D409H-GBA1 animals exhibited earlier loss of nigral neurons and aSyn neuropathology [103]. At variance, heterozygosity for N370S did not modify the phenotype of A30P-aSyn TG mice [113]. Elsewhere, viral delivery of N370S-GBA1, but not WT-GBA1, increased LC3 levels in the striatum of control mice and augmented aSyn release in A53T-aSyn TG mice [114].

These approaches reinforced the evidence that GCase and aSyn participate in a common pathway, with GCase modulating aSyn catabolism. While important at the biological level, it is still worth noting that these combinations are not present in patients, thus limiting their translational predictivity.

## A Critical Reappraisal of LRRK2 and GBA1 Animal Models

### Combining Insights Into Mechanistic Hypotheses

As is case for every animal model of neurodegenerative disorders, LRRK2- and GBA1-based genetic models do not meet the 3 requirements for the ideal preclinical animal model (construct, face and predictive validity [115]). Nevertheless, a profound knowledge of their characteristics allows the researchers to plan and design appropriate experimental paradigms and select the best model to answer the specific question at hand. The main task then is to combine answers from different models in a coherent and data-based conclusion with biological relevance.

For this purpose, it is useful to view the vast amount of literature on LRRK2 and GBA1 models (discussed in the previous sections, albeit not exhaustively) with the scope of gaining a larger picture of PD and put together the pieces of pathogenesis. I predict that this exercise, carried out by several labs in the world, will eventually lead to a prospective therapeutic strategy targeting the root of the disease. Accordingly, in this section I will attempt to reconcile data from LRRK2 and GBA1 animals toward 3 aspects of PD research: etiology; pathogenesis and progression; preclinical drug validation.

**Fig. 3:**
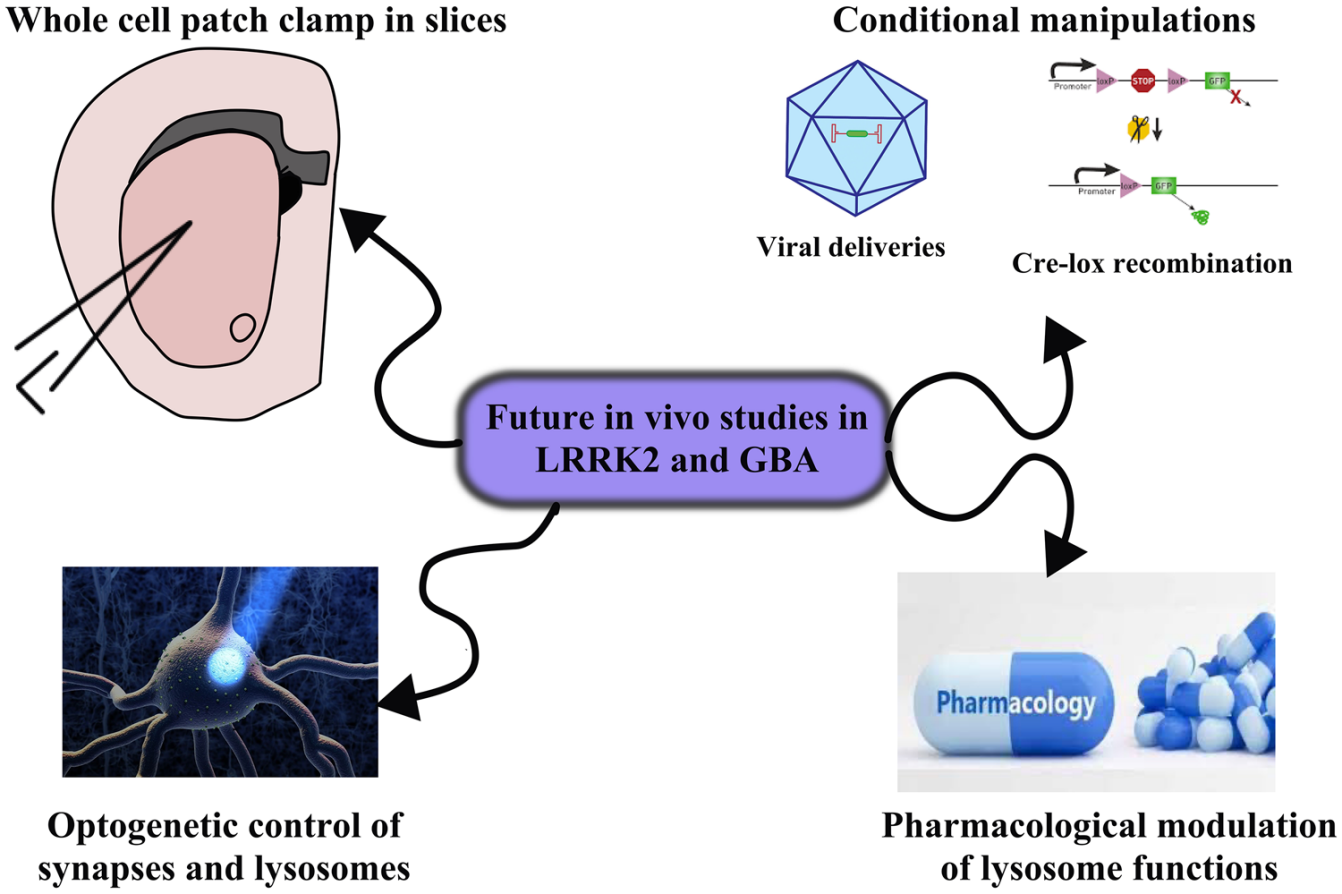
Proposed future in vivo studies in LRRK2 and GBA1 models. To advance our understanding of the functions of LRRK2 and GCase in the living brain, different experimental paradigms could be performed. To assess the synaptic roles of lysosome, and how LRRK2 and GCase might converge in this process, animal models can be utilized for electrophysiology in acute brain slices to measure neuronal and synaptic transmission in relevant brain areas (such as the striatum, depicted). The combination of LRRK2 and GBA1 manipulation could be performed to probe their functional interactions, using viral delivery approaches or crossing different models (with a special usefulness of conditional manipulations allowing temporal and spatial control). The biological connection between lysosomes and synapses could be explored with more accuracy exploiting the great advances in optogenetic control of both neuronal transmission and lysosome acidification. Lastly, lysosome function could be modulated pharmacologically in LRRK2, GBA1 and dual LRRK2-GBA1 animal models to acutely dissect the lysosomal contribution to phenotypes

The genetic aspect clearly favors the etiological relevance, which is best tackled by these models. In particular, LRRK2 mutations in its coding gene are recognized causes of familial disease and found in iPD patients as well [7, 8]. LRRK2 models bear an additional etiological advance, as the underlying biology also links familial PD and iPD. The pathogenic LRRK2 mutations cause an increase in its kinase activity that is detectable in mouse tissue [116]. Of note, the hyperactivation of LRRK2 kinase has also been reported in DA neurons of iPD patients, in the absence of any genetic alteration and in non LRRK2-based animal models [117]. These observations taken together deeply implicate LRRK2 biology (and not only genetics) in the pathogenic process of PD. This critical biological aspect similarly involves GBA1. Indeed, its dual relation with aSyn strongly suggest that GCase function is required to maintain a correct aSyn homeostasis. The biological implication probably surmounts the genetic one in the case of GBA1, as it is classified as a risk factor, albeit a strong one, and not a cause in itself. With these considerations in mind, I believe LRRK2 and GBA1 models (and likely their combination, see following section) will be more and more important to study the molecular and cellular pathways involved in disease onset (Fig. 3). This approach brings the invaluable advantage of neuronal and mammalian relevance, providing needed validation for findings obtained in cellular and/or invertebrate models. Specifically, describing detailed lysosomal pathways and their alterations in the brains of LRRK2 and GBA1 models will bring us closer to understanding how ALP regulates neuronal survival and “tips it over the edge” [118] in DA neurons and PD.

Directly relevant and temporally sequential to etiology and pathogenesis, lies modeling disease progression. This aspect finds more challenges as no LRRK2 nor GBA1 mice reproduce the full array of neuropathological and behavioral presentations of PD, and generally lack nigrostriatal degeneration. However, many models do display some characteristic reminiscent of PD. LRRK2 mice in particular are characterized by age-dependent changes in neuronal and synaptic functions [66, 119, 120]. This is probably of underestimated importance; indeed, if we posit that DA degeneration does begin at striatal axon terminals [121] and knowing that ALP plays a critical (and yet not completely elucidated) role at the synapse involving LRRK2 itself [14, 92] LRRK2 animals could be in the unique position of disclosing precise, pre-symptomatic alterations to neuronal biology. This improved temporal knowledge of events occurring well ahead of neuronal loss is fundamental to design true disease-modifying therapies.

Lastly, the ability of a model to predict the effect of a candidate drug in patients defines its preclinical usefulness. In this respect, LRRK2 and GBA1 are probably not yet capable of providing the full extent of characteristics required. However, LRRK2 kinase inhibitors do demonstrate target engagement in vivo [86, 116] and they protect animals from aSyn-induced neuropathology [22, 122], but these experiments were carried out in a “two-hit” approach as LRRK2 genetic models do not display aSyn accumulation or neurodegeneration. This evidence was nevertheless sufficient to initiate clinical trials. Similarly, GBA1 rodents do not present histological features of PD, as previously discussed, but were used to validate small molecule chaperones of GCase such as ambroxol, which is currently under clinical trial (NCT02941822) [123].

Altogether, these considerations lead to me to propose that the current state of the art in LRRK2 and GBA1 animal models might be best suited to validate mechanistic indications of pathogenesis, likely coming from cellular models, at the whole-organism level. Selected questions and/or pharmacological approaches can also be attempted, paying particular attention to the caveats of the model of choice to maximize results and, most of all, their reliability. With this in mind, I believe great progress can be made.

### Can We Further Ameliorate Modeling?

Amelioration of PD modeling in experimental animals has been a continuous effort and is still ongoing. The advent of genetics surely greatly improved the construct validity and the etiological fidelity. The lack of complete face validity though has fueled the use of a combination of different models. The treatment of genetic models with neurotoxins has seen utilization aimed at identifying increased sensitivity to induced neurodegeneration, and studying gene-environment interactions as disease triggers. A detailed description goes beyond the scope of this review article and the reader is directed elsewhere [124–126].

In the previous sections, I have discussed instances where LRRK2 and GBA1 models were combined mainly with aSyn manipulations (either via viral delivery or through crossbreeding). What the field has not yet seen is a combination of LRRK2 and GBA1 models themselves. Opposite to the other combinations, dual mutations in these genes do have etiological relevance as they have been described in PD patients and their clinical features evaluated retrospectively. The co-occurrence of LRRK2 p.G2019S and a GBA1 mutation is a rare event (~ 2% of ~ 1400 PD patients reported in 2 recent studies [127, 128]) and seems to additively increase the risk for disease, that is the chance of developing PD in the mutant carrier [127]. Oppositely from the effect on disease risk, phenotype-genotype correlations do not appear to indicate a difference in severity of motor symptoms, while the presence of the LRRK2 mutation has a controversial effect on cognitive and psychiatric decline, depending on the specific study [127, 128]. Thus, dual LRRK2-GBA1 mutations appear to exert different effects when considering the risk for PD and the severity of it, if and once manifested. The rarity of the dual presence of LRRK2 and GBA1 mutations in patients represents a challenge in the clear definition of clinical presentations, but from the biological point of view it is an important approach to consider in modeling. LRRK2 and GCase, as extensively discussed, have common and maybe partially overlapping functions in lysosome biology. In order to clearly dissect their contribution to lysosome function, their contemporary manipulation is required to clarify the pathway (i.e., KO, overexpression, pharmacological manipulation) and this has been extensively performed in cellular models. It would be helpful to conduct the same operations in rodent models (Fig. 3), crossing different genetic models to obtain, for instance, dual KI mice or a LRRK2 KI mouse on a GBA1 null background. The power of conditional genetic manipulations could be exploited at its fullest potential to restrict changes in the brain or specific neuronal populations. These models would be particularly apt to neurophysiological studies (Fig. 3), at in vivo, ex vivo (in acute brain slices) or primary culture levels. In this way, we could begin to unravel how lysosomes regulate neuronal function, synaptic transmission and plasticity, with the added opportunity to study their behavioral correlates in the same model in parallel experiments. Further, optogenetics and laser/optic fiber implantation would allow the concomitant correlation of these functions, also manipulating lysosomal pH via light stimulation [129] (Fig. 3). These objectives could also be reached combining genetic models with viral-mediated gene manipulations, to further control age and tissue factors, and enable both overexpression and silencing. The possible combinations of experimental groups would be numerous, allowing for several research questions to be addressed.

## Conclusions

The advancement in our knowledge of PD onset and progression, and the possibilities of nominating novel targets for therapy have been hugely boosted by the advent of genetics. The technological abilities in manipulating gene expression and function in living rodents matched these opportunities well. It allowed to study the functionality of these genes and the pathways in which they are involved with providing direct relevance to neurophysiology and a relationship to disease presentation.

LRRK2 and GBA1 are among the most intensely studied genes, and they directly point to a deep involvement of lysosome biology in the etiology of PD and the understanding of its development over time. The recent fascinating indications that lysosomes are not mere degradative vesicles but have functional signaling roles [42] and might impact synaptic function, independently or within ALP [92, 130], could be the missing link between early (synaptic) changes and late proteinopathy. The unequivocal temporal definition of these events will be, in my opinion, the next breakthrough in our fight against PD, and in advancing our understanding of the brain.

## Supplementary Information

Below is the link to the electronic supplementary material.

Supplementary file1 (PDF 498 KB)
